# Adult-Onset Still’s Disease (AOSD)—On the Basis of Own Cases

**DOI:** 10.3390/biomedicines12092067

**Published:** 2024-09-10

**Authors:** Małgorzata Wisłowska

**Affiliations:** Rheumatology Clinic, National Institute of Geriatrics, Rheumatology and Rehabilitation, 1 Spartanska Street, 02-637 Warsaw, Poland; malgorzata.wislowska@spartanska.pl; Tel.: +48-226-709-131

**Keywords:** adult-onset Still’s disease, macrophage activation syndrome (MAS), reactive hemophagocytic lymphohistiocytosis (HLH)

## Abstract

Introduction: Adult-onset Still’s disease (AOSD) is a rare chronic autoinflammatory condition characterized by a spiking fever, arthritis, a rash, hepatosplenomegaly, lymphadenopathy, leucocytosis, and hyperferritinemia. It is sometimes accompanied by life-threatening complications like macrophage activation syndrome/hemophagocytic lymphohistiocytosis (MAS/HLH). Treatment options for AOSD include glucocorticoids (GCs), immunosuppressive drugs, biological medications, and Janus kinase (JAK) inhibitors. The features that differentiate MAS/HLH from AOSD are: in MAS/HLH, a different type of fever, which is persistent, a sharp decrease in the number of leukocytes and thrombocytes, a further increase in the level of transaminases and ferritin, significant hepatosplenomegaly, lymphadenopathy, symptoms of the central nervous system (CNS), disseminated intravascular coagulation (DIC) and hemophagocytosis in the bone marrow. This study aimed to evaluate the course of AOSD, which results in MAS/HLD. Patients and methods: Nine AOSD patients, four of whom developed MAS/HLH, were treated at the Rheumatology Clinic in the Central Clinical Hospital of the Ministry of Interior Affairs from 1 January 2015 to 15 March 2020 and at the Rheumatology Clinic in the National Institute of Geriatric, Rheumatology and Rehabilitation from 1 September 2021 to 1 March 2024. Medical history, clinical data, demographic data, laboratory data, imaging data, Hscore, and treatment data were collected. Results: All the patients with MAS and an Hscore above 150 recovered. Discussion: MAS/HLH requires rapid diagnosis as well as treatment with methylprednisolone pulses, cyclosporine A, and etoposide. When comparing patients who developed MAS/HLH with those who did not, possible risk factors were identified: the presence of pregnancy (two cases) and an aggressive course of AOSD. The Hscore is a useful tool for identifying patients with MAS/HLH.

## 1. Introduction

Adult-onset Still’s disease (AOSD) was described by Eric Bywaters in 1970 as an inflammatory disease in young adults [1]. It is part of the spectrum of systemic-onset juvenile idiopathic arthritis (SJIA), which was described one hundred years ago by Sir John Still [2]. AOSD is a rare, difficult-to-diagnose systemic autoimmune inflammatory disease of unknown etiology, and is influenced by both genetic and environmental factors [3]. In Poland, the annual incidence rate of AOSD is 0.32 per 100,000, and the prevalence is 2.7 per 100,000 [4]. 

Still’s disease is characterized by a daily recurrent spiking fever that does not respond to treatment, and is accompanied by either two major criteria OR one major criterion and two minor criteria.

The major criteria include the following:

1. Transient (non-persistent) erythematous rash in the form of salmon-colored spots, most often located on the trunk and limbs; the rash is macular or slightly urticarial, and sometimes persistent red-brownish papules and plaques may be seen.
2. Arthritis (initially it may be very discreet, but then it causes significant damage to the musculoskeletal system).

The minor criteria include the following:

1. Generalized lymphadenopathy and/or hepatomegaly and/or splenomegaly.
2. Serositis.
3. Arthralgia lasting 2 weeks or more (in the absence of arthritis).
4. Leukocytosis (≥15,000/μL) with neutrophilia [5].

In Still’s disease, the course may vary in following forms:

1. A monocyclic course, characterized by a single episode followed by persistent remission.
2. A polycyclic course is characterized by recurrent flares and remissions.
3. A chronic course [6].

There are two subtypes of Still’s disease in adulthood:

1. Systemic AOSD is characterized by an increased concentration of the interleukin (IL)-1β, IL-18, interferon (INF)α/β, INFγ, IL-4, IL-10, hyperferritinemia, thrombocytopenia, and Natural-killer (NK) cell dysfunction. Clinical symptoms include fever, serositis, hepatitis, and MAS [6]. Monoarthritis is more prevalent in systemic AOSD, which correlates with the lower frequency of MAS among patients with polyarthritis, the chronic articular disease course, and the lower inflammatory markers [6]. MAS can present with wide range of hepatic dysfunction, from mild elevation of the transaminases to liver failure. [6]. Treatment should include IL-1, IL-18, or INFγ inhibitors [7].
2. Articular AOSD is associated with increased concentrations of IL-17, IL-23, tumor necrosis factor (TNF)α, and IL-6, as well as thrombocytosis. The clinical symptoms include arthritis with joint destruction. The recommended treatment includes IL-6 or TNFα or IL-17 inhibitors [7].

Table 1 presents different classification criteria for AOSD, e.g., Cush [8], Yamagushi [9], and Fautrel [10], and ILAR criteria for SJIA [11]. These criteria are very similar, but the most commonly used are Yamaguchi criteria [9], which have over 90% sensitivity. Notably, these criteria do not include increased ferritin levels, which have a high diagnostic value [9]. The Fautrel criteria include glycosylated ferritin, which further improves the diagnosis [10].

Table 2 shows the frequency of symptoms [12], typical joint involvement [8], and laboratory test abnormalities [13,14] in AOSD. Often, inflammation of the wrists and metacarpophalangeal (MCP) joints can lead to ankylosis. 

The diagnosis of Still’s disease requires a broad differential diagnosis, including the following:

- Severe viral infections (rubella, hepatitis, parvoviruses, coxsackie viruses, Epstein–Barr viruses [EBVs], cytomegaloviruses [CMVs], human immunodeficiency virus [HIV], severe acute respiratory syndrome coronavirus-2 [SARS-CoV-2], pediatric inflammatory multisystem syndrome temporally associated with SARS-CoV-2 [PIMS-TS]), or bacterial infections (bacterial endocarditis, meningococcal infection, brucellosis, yersiniosis, tuberculosis, Lyme disease, syphilis, as well as sepsis.
- Malignancies, including leukemia, lymphoma, neuroblastoma, and paraneoplastic syndromes.
- Rheumatic fever.
- Kawasaki disease.
- Other systemic connective tissue diseases, like systemic lupus erythematosus, idiopathic myositis, mixed connective tissue disease, rheumatoid arthritis, and vasculitis.
- Other autoinflammatory diseases related to IL-1, like Mediterranean fever, cryopyrin-associated periodic syndromes (CAPSs), TNF receptor-associated periodic fever syndrome (TRAPS), and mevalonate kinase deficiency (MKD) [5].

The differential diagnosis should also include the following: sarcoidosis, reactive arthritis, Schnitzler syndrome, Sweet syndrome, and hypersensitivity syndrome on a drug [5]. 

Complications of Still’s disease may include the following: pleurisy, pericarditis, cardiac tamponade, myocarditis, endocarditis, disseminated intravascular coagulation (DIC), pulmonary fibrosis, pulmonary hypertension, vesicular bleeding, kidney insufficiency, neuropathy, aseptic meningitis, cataracts, fulminant hepatitis, thrombotic thrombocytopenic purpura, and MAS/HLH [5,8,14].

Symptoms of MAS include persistent high fever, hepatosplenomegaly, generalized lymphadenopathy, CNS symptoms (headaches, disturbances of consciousness, coma), DIC, respiratory, circulatory and urinary insufficiency, and a rash [15]. Laboratory tests show relative cytopenia, which might be in the normal range, involving ≥ 2 cell lines, as well as leucopenia, lymphopenia, anemia, thrombocytopenia, increased AST activity, hypertriglyceridemia, hypofibrinogenemia, hyperferritinemia, decreased albumin levels, increased lactate dehydrogenase (LDH) activity > 900 U/L, decreased or absent NKs activity, and hemophagocytosis in the bone marrow [15]. According to Ravelli et al. [15], the following values confirming MAS were observed: ferritin > 684 ng/mL, platelet count ≤ 181 × 10^9^/L, AST > 48 U/L, triglycerides > 156 mg/dL, and fibrinogen ≤ 360 mg/dL.

Symptoms of MAS include persistent high fever, hepatosplenomegaly, generalized lymphadenopathy, CNS symptoms (headaches, disturbances of consciousness, coma), DIC, respiratory, circulatory and urinary insufficiency, and a rash [15]. Laboratory tests show relative cytopenia, which might be in the normal range, involving ≥ 2 cell lines, as well as leucopenia, lymphopenia, anemia, thrombocytopenia, increased AST activity, hypertriglyceridemia, hypofibrinogenemia, hyperferritinemia, decreased albumin levels, increased lactate dehydrogenase (LDH) activity > 900 U/L, decreased or absent NKs activity, and hemophagocytosis in the bone marrow [15]. According to Ravelli et al. [15], the following values confirming MAS were observed: ferritin > 684 ng/mL, platelet count ≤ 181 × 10^9^/L, AST > 48 U/L, triglycerides > 156 mg/dL, and fibrinogen ≤ 360 mg/dL.

Symptoms of MAS include persistent high fever, hepatosplenomegaly, generalized lymphadenopathy, CNS symptoms (headaches, disturbances of consciousness, coma), DIC, respiratory, circulatory and urinary insufficiency, and a rash [15]. Laboratory tests show relative cytopenia, which might be in the normal range, involving ≥ 2 cell lines, as well as leucopenia, lymphopenia, anemia, thrombocytopenia, increased AST activity, hypertriglyceridemia, hypofibrinogenemia, hyperferritinemia, decreased albumin levels, increased lactate dehydrogenase (LDH) activity > 900 U/L, decreased or absent NKs activity, and hemophagocytosis in the bone marrow [15]. According to Ravelli et al. [15], the following values confirming MAS were observed: ferritin > 684 ng/mL, platelet count ≤ 181 × 10^9^/L, AST > 48 U/L, triglycerides > 156 mg/dL, and fibrinogen ≤ 360 mg/dL.

Both HLH/MAS and SJIA/AOSD present with a high fever, hepatosplenomegaly, lymphadenopathy, liver dysfunction, hyperferritinemia, and coagulopathy. 

The primary distinction is the level of neutrophilia, which increases in AOSD/SJIA and decreases in HLH [5,8]. MAS indicators include an increase in ferritin levels, a sharp decrease in the number of leukocytes and an increase in the level of transaminases.

IL-18 affects NKs and the cluster of differentiation (CD)8+ cells, leading to excessive secretion of INFy and hemophagocytic transformation of macrophages [16]. IL-1, the inflammasome receptor, and the Nod-like receptor (NLR) 3 play a similar role [17]. 

Hematologists use the “Hscore” to diagnose HLH. This score assesses the likelihood of HLH in a patient. An Hscore of ≤90 HLH may indicate that HLH can be excluded, while a value ≥250 suggests a greater than 99% probability of HLH [18]. Table 3 presents the Hscore criteria for the diagnosis of HLH.

The purpose of this study was to evaluate the progression of AOSD, which can result in MAS/HLD, a severe complication of AOSD. An intriguing open question remains regarding cases that are more susceptible to this serious complication, and this paper attempts to answer it and to offer the patient a successful treatment. 

## 2. Materials and Methods

Nine patients with severe AOSD were treated at the Rheumatology Clinic in the Central Clinical Hospital of the Ministry of Interior Affairs Warsaw, Woloska 137 street, Poland between 1 January 2015 and 15 March 2020 (Head of Clinic prof. M. Wislowska MD, PhD) and at the Rheumatology Clinic in the National of Institute of Geriatric, Rheumatology and Rehabilitation Warszaw, Spartanska 1 street, Poland between 1 September 2021 and 1 February 2024 (Head of Clinic prof. M. Wislowska MD, PhD). The demographic data, medical history, clinical data, laboratory data, and imaging data were collected. The medical treatments, side effects, and outcomes were recorded chronologically. All data were collected at the time of the AOSD diagnosis and at the time of the MAS diagnosis. Hscores were retrospectively calculated [18]. Table 4 presents nine cases of AOSD, detailing their age, sex, symptoms, and laboratory data, which confirmed the AOSD diagnosis and the medical treatment pursued. During hospitalization, four cases developed MAS/HLH syndrome; among these were two pregnant patients.

## 3. Ethics

Ethical approval was obtained from the institutional review board [N. KBT-4/3/2024]. All the patients signed informed consent forms. 

## 4. Results

In four cases, complications like MAS/HLH were observed. The symptoms, laboratory data confirming MAS/HLH syndrome, and the treatment of these patients are presented in Table 5. The differences between patients with or without MAS are the following: hepatomegaly, a rapid decrease in the level of thrombocytes in one case, and a rapid increase in the level of ferritin. In all patients who developed MAS, a change in the fever pattern from spiking to a persistent high fever was observed. All patients had hepatomegaly and elevated transaminases, with one case exceeding 2000 IU/L. A decrease in erythrocytes was observed in two patients, leukopenia was observed in one patient, and thrombocytopenia was observed in three patients. Furthermore, all patients presented with elevated ferritin levels. The Hscores for all patients exceeded 150 points and are presented in Table 6. As treatment, all patients received glucocorticosteroids in pulses; additionally, three patients received cyclosporine A and IVIG, two received tocilizumab, and one underwent plasmapheresis. All the patients were treated immediately and therefore, they were successfully cured. 

## 5. Discussion

A dangerous complication of SJIA and AOSD is MAS/HLH, a potentially life-threatening complication characterized by a systemic inflammatory reaction due to an uncontrolled and dysfunctional immune response, which results in a massive hypersecretion of pro-inflammatory cytokines [15]. The basis of this syndrome is the uncontrolled expansion of T cells and macrophages with a dysfunction of NK and cytotoxic CD8+ cells, as well as a reduced expression of perforin and granzyme H, which results in an escalation of the production of the INFy and pro-inflammatory cytokines as well as macrophage hemophagocytosis [15]. 

SJIA and AOSD are categorized as polygenic autoinflammatory diseases. There are distinct mechanisms that differentiate autoimmune diseases (“autoimmunities”) from autoinflammatory diseases (“autoinflammation”). “Autoimmunity” involves a dysregulation of adaptive immunity, resulting in an aberrant response of the dendritic cells, B cells, and T cells to their own tissues, which leads to a breakdown of tolerance and immunological responses to self-antigens, accompanied by the production of autoantibodies. In contrast, “autoinflammation” refers to the dysregulation of an innate immunity that triggers excessive activation of phagocytes, including macrophages and neutrophils, in response to stress factors such as PAMSs (pathogen-associated molecular patterns) and DAMPs (danger-associated molecular patterns), resulting in tissue damage [19]. While autoimmune diseases are easily diagnosed by the presence of antibodies, there are no specific biomarkers for diagnosing systemic autoinflammatory disorders (SAIDs) [20]. Still’s disease is a polygenic non-familial SAID that responds to treatment with IL-1 blockers. Infectious agents such as bacteria and viruses, including Yersinia enterocolitica, Mycoplasma pneumoniae, rubella, and parvovirus B19, can act as triggers [21]. Examining the liver enzymes and cytokines as biomarkers will contribute to the differential diagnosis of infection-associated diseases, e.g., viral hepatitis or COVID-19, but the specificity issues of these biomarkers should be carefully considered [22,23].

Toll-like receptors (TLRs) play crucial roles in detecting viral nucleic acids, a key type of viral PAMP and DAMP, and initiate signaling pathways that lead to the activation of interferon regulatory factor 3 (IRF3) and nuclear factor kappa B (NF-κB) [24]. The tripartite-motif protein-56 (TRIM56) enhances the induction of the type I interferon (INF) response through the TLR3 pathway by boosting IRF3 activation [25]. The depletion of chaperone proteins, like the glucose-regulated protein 78 kDa (GRP78), hampers the establishment of an antiviral state mediated by TLR3 [26]. Upon binding the PAMP molecules to the TLRs in macrophages and neutrophils, the activated Nod-like receptor (NLR) 3 forms a complex called an inflammasome that activates caspase 1 or 11. The caspases in the inflammasome lead to an overproduction of IL-1β, TNF, IL-6, IL-8, IL-17, and IL-18, triggering a “cytokine storm”. Additionally, alarmins, such as the S100 and S100A12 proteins, activate the macrophages and neutrophils. A disruption of the anti-inflammatory mechanisms and a deficiency in regulatory T cells, NKs, cells and IL-10 occur alongside these processes. The genetic background is also a significant factor [5]. Genetic factors in SJIA include the presence of the human leukocyte antigen (HLA)—Bw35, DR2, DR4, DR5, and Dw7— as well as the presence of macrophage migration inhibitory factors (MIFs), IL6, IL18, TNFα polymorphisms, and, in AOSD, HLA- Bw35, DR2, DR4, DRB1, Dw7, and MIFs, which are IL18 polymorphisms [5].

In AOSD, the adaptive immune system plays a minor role. Markers such as macrophage colony-stimulating factor (M-CSF) and INF-γ, calprotectin, MIFs, and intercellular adhesion molecule (ICAM)-1, which activates monocytes, correlate with disease activity [27]. In SJIA, there is a disturbed regulation of innate immune receptors such as NLR, TLR5, and TLR4. TLR4 recognizes S100 proteins. High concentrations of circulating S100 proteins occur in the active phase of the disease, and the S100-A8/S100-A9 complex, through TLR4, triggers the secretion of IL-1β [27]. IL-1β likely initiates AOSD by causing systemic inflammation and stimulating damage to cartilage and bone [27]. High levels of IL-18 are found in the serum, synovium, lymph nodes, and liver of patients with SJIA and AOSD. IL-18 induces a T helper (Th) 1 response and affects the secretion of INF-γ by cytotoxic CD8+ and NKs and it has been suggested that it plays a major role in the pathogenesis of reactive hemophagocytic lymphohistiocytosis [28]. IL-6 and TNFα also contribute to the pathogenesis of AOSD, although TNFα levels do not correlate with disease activity [21]. IL-17 increases inflammation, stimulates the production of chemokines that recruit neutrophils, enhances granulopoiesis and myelopoiesis, and plays an important role in the development of arthritis in AOSD [29]. In SJIA and AOSD, there are not only pro-inflammatory mechanisms, but also defects in immunoregulation [30]. 

High serum ferritin levels indicate macrophage activation and correlate with the disease activity in AOSD, serving as a sensitive marker [13]. Besides ferritin, glycosylated ferritin (GF) is crucial for diagnosis. Normally, it accounts for over half the ferritin level. However, its level drops to 20–50% in inflammatory diseases and falls below 20% in AOSD [31,32]. 

Procalcitonin, typically a marker of severe systemic infection, may rise in patients with active AOSD, thus complicating the differentiation between acute infections and AOSD flare [5]. Additionally, increases in IL-1, IL-6, IL-18, TNF, and INFγ have been observed in AOSD [27]. 

These processes are still not fully understood, especially in AOSD patients who develop HLH/MAS. According to a study by Javaux et al. [33], which analyzed 206 patients with Still’s disease across nine regional French centers from January 2001 to March 2021, 20 of the patients developed MAS. Patients with MAS were more likely to exhibit hepatosplenomegaly and neurological symptoms. Similarly, in my small cohort, I observed hepatosplenomegaly in my patients. Javaux et al. [33] also noted that extreme hyperferritinemia at the onset of Still’s disease is a prognostic factor for the development of MAS. Not all of my MAS patients exhibited extreme hyperferritinemia. 

An important paper discussed the clinical and laboratory features associated with macrophage activation syndrome in Still’s disease based on data from the international Autoinflammatory Disease Alliance (AIDA) Still’s Disease Registry [34]. Of the 414 patients with Still’s disease included in the study, 39 of them developed MAS. A multivariate analysis showed direct associations between MAS, hepatomegaly, and monoarthritis, while a normal platelet count or thrombocytosis appeared to be protective [34].

Nonsteroidal anti-inflammatory drugs (NSAIDs) are used as the first-line drugs in the treatment of AOSD before diagnosis is established. After diagnosis, GCs should be administered. The best response is observed with an initial dose of 0.8–1 mg/kg of prednisone, to which approximately 60% of patients respond, or after pulsatile administration of methylprednisolone at a dose of 500–1000 mg/day for 2–3 days. Synthetic disease modifying anti-rheumatic drugs (DMARDs), such as methotrexate in a dosage of up to 25 mg/week (approximately 70% effectiveness), along with other options like azathioprine, leflunomide, cyclosporine A, and tacrolimus, are considered. Patients with AOSD should not be treated with sulfasalazine [5,14,35].

Treatment of AOSD depends on the clinical picture. In the case of arthralgia or arthritis predominance, TNF inhibitors are preferred as second-line medications, but they are ineffective for HLH [36]. In cases of fever, rash, pharyngitis, and serositis, leukocytosis IL-1β inhibitors (anakinra, canakinumab, rilonacept) are the treatment of choice [37,38,39]. Anakinra, an interleukin 1 receptor antagonist, is administered at a dose of 2 mg/kg to 4 mg/kg of body weight, with 100–200 mg given subcutaneously (SC) daily [37]; canakinumab, an anti-interleukin 1 antibody, is given at a dose of 4 mg/kg of body weight every 4 weeks, with a maximum of 300 mg given SC every 4 weeks [38]; rilonacept, a fusion protein composed of the human extracellular domain of the IL-1 receptor and the Fc fragment of the IgG1 immunoglobulin, is administered SC at a dose of 100–320 mg every week [39]. In clinical studies, complete remission was observed in 73% of cases after anakinra treatment, in 55% after rilonacept treatment, and 75% after canakinumab treatment [37,38,39]. Tadekinig alfa, an IL-18 binding protein inhibitor, is given subcutaneously at a dose of 80–160 mg every week [16]. In cases of HLH/MAS, hyperferritinemia, and liver involvement, the IL-1 inhibitors or the IL-6 receptor inhibitor (Tocilizumab) [40] as well as the JAK inhibitors (Tofacitinib, Baricitinib) [41,42] are very useful. Approximately 20–25% of patients with AOSD are treated with biological DMARDs [36,37,38,39,40].

Tocilizumab, an interleukin 6 receptor antagonist, is administered IV and SC at a dose of 162 mg every week or 8 mg/kg of body weight every 2 weeks. It is effective in treating arthritis and systemic symptoms [40]. 

The risk of MAS in patients with AOSD treated with IL-1 and IL-6 inhibitors shows that the incidence of MAS is significantly higher in patients treated with tocilizumab (*p* = 0.01) [43]. 

Some AOSD patients may be treated with rituximab (1 g given IV on days 1 and 15, and subsequent cycles every 6 months) [44] or cyclophosphamide [45]. Intravenous immunoglobulins (IVIG) administered at 0.4g/kg to 2 g/kg of body weight for 2–5 days a month have shown potential efficiency [46]. Abatacept has been used in the treatment of AOSD in some patients [47]. The inhibitor IL-17 is under preliminary observations [48].

MAS can lead to progressive multi-organ failure and, eventually, a fatal outcome [48]. According to the 2022 EULAR/ACR guidelines for the early stages of diagnosis and the management of suspected hemophagocytic lymphohistiocytosis/macrophage activation syndrome (HLH/MAS), HLH and MAS are life-threatening systemic hyperinflammatory syndromes characterized by a fever, elevated ferritin levels, low blood cell counts, DIC, hepatitis, CNS inflammation, and a high risk of progression to multiple organ dysfunction, shock, and even death [49]. The term HLH originally described a pathological condition in young children as a “primary” genetic defect. MAS, on the other hand, is a complication seen in rheumatic diseases such as SJIA or systemic lupus erythematosus (SLE). The task force agreed to refer to the entire spectrum of primary or secondary HLH as “HLH/MAS”. This condition can occur in any age group and develops in the context of infectious, malignant or rheumatological diseases, or due to the congenital errors of immunity that predispose people to hyperinflammation. However, early recognition of MAS/HLH is often challenging yet crucial [49]. 

MAS/HLH requires quick diagnosis and immediate intensive treatment with methylprednisolone pulses, cyclosporine A, and etoposide. The neutralization of the IL-18/INFγ axis is a promising new therapeutic target in MAS/HLH [16]. Emapalumab, an INFy blocker, is a recombinant human IgG1 monoclonal antibody to gamma interferon, which inhibits its binding to the cell surface interferon receptors and the subsequent activation of the intracellular pro-inflammatory signaling pathways [50]. Gamma interferon levels are elevated in HLH patients. Emapalumab was approved for use in HLH in the United States in 2018. It is available as a solution in single-dose vials of 10 mg in 2 mL or 50 mg in 10 mL (5 mg/mL). The recommended starting dose is 1 mg/kg as an intravenous infusion twice per week. Doses can be adjusted based on clinical and laboratory results and therapy can be continued until hematopoietic cell transplantation is performed, unacceptable toxicity occurs, or it is no longer deemed necessary.

The most critical factor for the effective treatment of MAS/HLH is the immediate diagnosis of this syndrome. Comparing patients who developed MAS/HLH with those without these complications, an additional factor that determined a more severe disease course was the concomitant pregnancy in patients N.1 and N.8. Patient N.7 had a very long and aggressive course of the disease and had previously suffered one incident of MAS/HLH. However, patient N.9 had a very aggressive onset of the disease and developed MAS, which resolved after a treatment of high doses of GCs. In the cases I presented, MAS/HLH syndrome was diagnosed immediately, supporting the effectiveness of aggressive treatment. A limitation of this study is the small number of patients; however, the disease is very rare. The Polish population is about 38 million people, but the annual incidence rate of AOSD in Poland is 0.32 per 100,000 people, and the incidence of MAS in these patients is even rarer [4]. 

## 6. Conclusions

AOSD is a rare systemic autoinflammatory disease that can have a severe course and is accompanied by a wide spectrum of symptoms and complications, including MAS/HLH. Activation of the innate immune response and overproduction of several inflammatory cytokines, such as IL-1, IL-6, and IL-18, play key role in its pathogenesis. A high ferritin concentration is most commonly observed in the cases of AOSD or HLH. An immediate diagnosis and early, aggressive treatment of MAS/HLH during the course of AOSD can ensure effective management of this life-threatening complication.

## Figures and Tables

**Table 1:** Classification criteria for AOSD and the revised definition of the International League of Associations for Rheumatology (ILAR) diagnostic criteria for SJIA.

1987 Cush Criteria [8]	1992 Yamaguchi Criteria [9]	2002 Fautrel Criteria [10]	2004 ILAR Criteria [11]
2 points each: Quotidian fever > 39 °C Evanescent rash White blood cells (WBC) > 12,000/μL and erythrocytes sedimentation rate (ESR) > 40 mm/1h Negative antinuclear antibody (ANA) and rheumatoid factor (RF) Carpal ankylosis	Major criteria: Fever > 39 °C, intermittent, one week or longer Arthralgia or arthritis ≥ 2 weeks Characteristic rash WBC > 10,000/μL with > 80% granulocytes	Major criteria: Spiking fever ≥ 39 °C Arthralgia Transient erythema Pharyngitis ≥80% granulocytes Glycosylated ferritin ≤ 20%	Major criteria: Arthritis in at least one joint Fever > 2 weeks, daily for at least 3 days
1 point each: Onset age > 35 years Arthritis Sore throat Reticuloendothelial system involvement or liver function test (LFT) abnormality Serositis Cervical or tarsal ankylosis	Minor criteria: Sore throat Lymphadenopathy Hepatomegaly or splenomegaly Abnormal liver function tests Negative ANA and RF	Minor criteria: Maculopapular rash Leucocytes ≥ 10,000/μL	Minor criteria: Evanescent erythematous rash Generalized lymph node enlargement Hepatomegaly Splenomegaly Serositis
Exclusion criteria: None	Exclusion criteria: Infections, malignancy, other rheumatic diseases that mimic AOSD	Exclusion criteria: None	Exclusion criteria: Other forms of JIA must be excluded
Algorithm: Probable AOSD: 10 points during 12 weeks of observation Definite AOSD: 10 points with 6 months observation	Algorithm: 5 criteria: at least 2 major ones AND no exclusion criteria	Algorithm: 4 major criteria OR 3 major with 2 minor ones	Algorithm: All major criteria AND at least 1 minor criterion
Sensitivity: Not applicable Specificity: Not applicable	Sensitivity: 96.2% Specificity: 92.1%	Sensitivity: 80.6% Specificity: 98.5%	Sensitivity: Not applicable Specificity: Not applicable

**Table 2:** Frequency of symptoms, joint involvement, and laboratory test abnormalities in AOSD.

Symptoms	Frequency in %	Involved Joints	Frequency in %	Laboratory Test Abnormalities	Frequency in %
Fever Arthralgia/arthritis Skin rashes Myalgia Sore throat Lymphadenopathy Hepatomegaly Splenomegaly Abdominal pain Pleuritis or pericarditis	85–100 73–95 68–81 44–65 53–62 31–61 7–22 5–67 14–18 20–25	Knee Wrist Ankle Proximal interphalangeal (PIP) Elbow Shoulder MCP Metatarsophalangeal (MTP) Hip Distal interphalangeal (DIP) Temporomandi-bular	84 74 57 47 50 43 34 19 14 18 8	Increased ESR and CRP Leukocytosis > 10,000/mm^3^ Neutrophil polymorphonuc-lear count ≥ 80% Anemia ≤ 10g/100 mL Thrombocytosis > 400,000/mm^3^ Abnormal liver function results Increased ferritin concentration Reduced glycosylated ferritin concentration ANA absence RF absence	99 89–94 88–93 50–75 62 43–76 69–93 80–90 92 93

**Table 3:** Hscore for the diagnosis of HLH [18].

Variable	Variable	Number of Points
Temperature	<38.4 °C 38.4–39.4 °C >39.4 °C	0 33 49
Organomegaly	None Hepatomegaly or splenomegaly Hepatomegaly and splenomegaly	0 23 38
Cytopenia	One lineage Two lineages Three lineages	0 24 34
Triglycerides (mmol/L)	<1.5 1.5–4.0 >4.0	0 44 64
Fibrinogen (g/L)	>2.5 ≤2.5	0 30
Ferritin (ng/mL)	<2000 2000–6000 >6000	0 35 50
Serum aspartate aminotransferase (IU/L)	<30 ≥30	0 19
Hemophagocytosis on bone marrow aspirate	No Yes	0 35
Known immunosuppression	No Yes	0 18

**Table 4:** Cases of Still’s disease.

No. of Case	Sex/Age	Criteria of Diagnosis	Complications	Treatment
1	F/34 18-week HBD	Intermittent fever > 39 °C, arthralgia, typical rash, leucocytes > 28,000 μg/L, granulocytes 84.5%, sore throat, ferritin 2217 ng/mL	MAS/HLH Th5 compressive fracture	GCs, IVIG, cyclosporin A (CsA), methotrexate (MTX), Etanercept, Tocilizumab
2	M/24	Arthritis (wrists, knee), sore throat, maculopapular rash, leucocytes > 18,300 μg/L, 84.4% granulocytes, ferritin 2006 ng/mL		GCs, MTX
3	F/30	Intermittent fever > 39 °C, arthralgia, typical rash, leucocytes > 20,100 μg/L, granulocytes 83%, sore throat, ferritin 2067 ng/mL		GCs, MTX
4	F/31	Intermittent fever > 39 °C, arthralgia, typical rash, leucocytes > 15,700 μg/L, granulocytes 82% sore throat, ferritin 4405 ng/mL		GCs, MTX
5	M/41	Intermittent fever > 39 °C, arthralgia, typical rash, leucocytes > 13,870 μg/L, granulocytes 83%, ferritin 16,016 ng/mL		GCs
6	F/28	Intermittent fever > 39 °C, arthritis (wrists), typical rash, lymphadenopathy, leucocytes > 14,720 μg/L, granulocytes 83.3% sore throat, ferritin 799 ng/mL		GCs, MTX
7	M/19	Intermittent fever > 39 °C, arthritis (wrists), typical rash, leucocytes > 20,980 μg/L, granulocytes 89.4% sore throat, ferritin 4405 ng/mL	MAS/HLH	GCs, CyC, intravenous immunoglobulin (IVIG), Tocilizumab
8	F/26, 22-week HBD	Intermittent fever > 39 °C, arthritis (wrists, knees), typical rash, sore throat, pericarditis, pleuritis, leucocytes > 21,030 μg/L, granulocytes 88.6%, ferritin 21,140 ng/mL, hepatomegaly. Previously, at age 10, the patient suffered one incident of MAS/HLH	MAS/HLH	GCs, CsA, IVIG, etanercept, MTX
9	F/30	Intermittent fever > 39 °C, arthritis (wrists, feet), slight urticarial rash, lymphadenopathy, hepatosplenomegaly, leucocytes > 8670 μg/L, ferritin 885 ng/mL	MAS/HLH	GCs

**Table 5:** Cases with AOSD who developed MAS/HLH syndrome.

Clinical and Laboratory Feature	Case 1 Female/34 Years 18 Week HBD	Case 7 Male/19 Years	Case 8 Female/26 Years 22 Week HBD	Case 9 Female/30 Years
Persistent fever	>39.5 °C	>39.5 °C	>39.5 °C	>39.5 °C
Hemorrhagic rash	Absent	Present	Absent	Absent
Hepatosplenomegaly	Present	Present	Only hepatomegaly	Present
Erythrocytes (μg/L)	26,000	Within normal range	28,500	Within normal range
Lymphocytes (μg/L)	Within normal range	Within normal range	Within normal range	2.46
Thrombocytes (μg/L)	106,000	55,000	159,000	Within normal range
Ferritin (ng/mL)	6217	79,187	36,985	1062
AspAT (IU/L)	2423	138	453	66
Fibrinogen (g/L)	1.98	0.88	4.0	1.67
Triglycerides (mg/dL)	215.2	335.4	275	138.5

**Table 6:** The value of Hscore patients with MAS/HLH syndrome.

Parameters of Hscore	Case 1 Female/34 Years 18 Week HBD	Case 7 Male/19 Years	Case 8 Female/26 Years 22 Week HBD	Case 9 Female/30 Years
Immunosuppression	18	18	18	18
Temperature	49	49	49	49
Organomegaly	38	38	23	38
Cytopenias	24	0	24	0
Ferritin	50	50	50	0
Fibrinogen	30	30	0	30
Triglycerides	44	64	44	0
Transaminase	19	19	19	19
Total score	272	268	227	154
Treatment	GCs, CyS, IVIG, tocilizumab	GCs, CyS, IVIG, tocilizumab	GCs, CyS, IVIG, plasmapheresis	GCs

## Data Availability

The original contributions presented in the study are included in the article, further inquiries can be directed to the corresponding author.
